# Influence of Maturation Status on Eccentric Exercise-Induced Muscle Damage and the Repeated Bout Effect in Females

**DOI:** 10.3389/fphys.2017.01118

**Published:** 2018-01-05

**Authors:** Ming-Ju Lin, Kazunori Nosaka, Chih-Chiao Ho, Hsin-Lian Chen, Kuo-Wei Tseng, Sébastien Ratel, Trevor Chung-Ching Chen

**Affiliations:** ^1^Department of Physical Education, Health and Recreation, National Chiayi University, Chiayi, Taiwan; ^2^Centre for Exercise and Sports Science Research, School of Medical and Health Sciences, Edith Cowan University, Joondalup, WA, Australia; ^3^Department of Physical Education, National Taiwan Normal University, Taipei City, Taiwan; ^4^Department of Exercise Health Sciences, University of Taipei, Taipei City, Taiwan; ^5^Université Clermont Auvergne, Laboratoire des Adaptations Métaboliques à l'Exercice en conditions Physiologiques et Pathologiques (AME2P), Clermont-Ferrand, France

**Keywords:** maturation, muscle strength, delayed onset muscle soreness, lengthening contractions, proprioception

## Abstract

This study compared changes in indirect muscle damage markers, proprioception and arterial stiffness after elbow flexor eccentric exercise between pre-pubescent (9–10 y), pubescent (14–15 y), and post-pubescent (20–24 y) healthy, untrained females (*n* = 13/group). The maturation of the participants was confirmed by the hand bone age. All participants performed two bouts of 30 sub-maximal eccentric contractions (EC1, EC2) using a dumbbell set at 60% of pre-exercise maximal voluntary isometric elbow flexion strength at 90°. Changes in maximal voluntary concentric contraction (MVC) torque, muscle soreness (SOR), plasma creatine kinase activity, proprioception (position sense, joint reaction angle) and arterial stiffness (carotid-femoral pulse-wave velocity: cfPWV) before to 5 days after EC1 and EC2 were compared among groups by a mixed-design two-way ANOVA. Pre-exercise MVC torque and cfPWV were smaller (*P* < 0.05) for the pre-pubescent (MVC: 10.0 ± 0.9 Nm, cfPWV: 903 ± 60 cm/s) and the pubescent (14.3 ± 1.1 Nm, 967 ± 61 cm/s) than the post-pubescent (19.1 ± 1.4 Nm, 1,103 ± 73 cm/s). Changes in all variables after EC1 were smaller (*P* < 0.05) for the pre-pubescent (e.g., MVC at 1 d post-exercise: −10 ± 6%, peak SOR: 5 ± 2 mm) than the pubescent (−15 ± 9%, 12 ± 6 mm) and the post-pubescent (−25 ± 7%, 19 ± 13 mm). After EC2, changes in all variables were smaller (*P* < 0.05) than those after EC1 for all groups (e.g., MVC at 1 d post-exercise, pre-pubescent: −4 ± 6%, pubescent: −9 ± 4%, post-pubescent: −14 ± 5%; peak SOR: 3 ± 2, 7 ± 3, 11 ± 6 mm), but the magnitude of the repeated bout effect was not different (*P* > 0.05) among the groups. These results show that the extents of muscle damage, and proprioception and arterial stiffness changes after eccentric exercise are greater at later stages of maturation, but the repeated bout effect is not affected by maturation.

## Introduction

Unaccustomed eccentric exercise induces muscle damage that is commonly indicated by prolonged decreases in muscle function, delayed onset muscle soreness (DOMS), and increased muscle proteins such as creatine kinase (CK) and myoglobin (Mb) in the blood (Hyldahl and Hubal, 2014; Goodall et al., 2017; Warren et al., 2017). Many studies have investigated exercise-induced muscle damage, but only a limited number of the studies have focused on the responses of children and adolescents to eccentric exercise.

To the best of our knowledge, six studies have reported muscle damage in children in comparison to adults after either downhill running (Webber et al., 1989), bench press (Soares et al., 1996), plyometric jumps (Marginson et al., 2005; Gorianovas et al., 2013), or maximal isokinetic eccentric contractions of the elbow flexors (Chen et al., 2014) and knee extensors (Deli et al., 2017). All of these studies showed that muscle damage was less in children than in adults. However, five of them used males for the comparison, and only one study (Webber et al., 1989) included females.

Webber et al. (1989) showed that pre-pubescent boys had a significantly greater increase in serum CK activity following a single bout of downhill running when compared to pre-pubescent girls, but the increases in serum CK activity were similar in children and adults when values were adjusted for body mass. In the study, other markers of muscle damage such as the loss of muscle function and DOMS were not included. Thus, limited information is available for the effects of growth or maturation on muscle damage in females.

Chen et al. (2014) have reported that muscle damage is greater for adult men than adolescent and pre-adolescent boys, and pre-adolescent boys showed only minor muscle damage after maximal eccentric exercise of the elbow flexors. However, the study set the shoulder joint at 45° flexion during the eccentric exercise, thus it was possible that biceps brachii muscle of the boys operated at shorter muscle lengths, resulting in less muscle damage (Chen et al., 2014). It is necessary to confirm the findings of the previous study by minimizing the possible influence of the shoulder joint angle. This may be possible to set the shoulder joint at 0° flexion in an eccentric exercise of the elbow flexors.

O'Brien et al. (2010a) compared changes in mechanical properties of human patellar tendon between men, women (close to 30 y), boys and girls (close to 9 y). They reported that the tendon stiffness was 94% greater in men than boys, and 84% greater in women than girls, and Young's modulus was 99% greater in men than boys, and 66% greater in women than girls. They concluded that the mechanical stiffness of the tendon increased with maturation due to an increased Young's modulus, and in females due to a greater increase in tendon cross-sectional area than tendon length. It is interesting to note that the effect of maturation on tendon properties is different between females than males. Chen et al. (2011) found that the magnitude of muscle damage induced by eccentric exercise was greater for less flexible muscle than flexible muscle. Given that the tendon stiffness is a determinant of muscle flexibility (Kawakami et al., 2008), it is possible that the magnitude of the difference in muscle damage between children and young adults is different between females than males. Moreover, some studies showed a difference in the magnitude of muscle damage between adult males and females such that muscle damage was less for females than males, due to the possible protective effect of estrogen on plasma membrane (Dannecker et al., 2003; Sewright et al., 2008; Sipaviciene et al., 2013). If this is the main reason for the sex difference in eccentric exercise-induced muscle damage, it is possible that no sex difference exists for the muscle damage in pre-pubescent children. However, the profiles of eccentric exercise-induced muscle damage in pre-pubescent and pubescent females are not known. Thus, it is of importance and interest to compare between pre-pubescent, pubescent and post-pubescent females for several aspects of muscle damage.

When muscle damage is induced, proprioception is affected such that position sense (PS) or joint reaction angle (JRA) were reduced after maximal eccentric contractions of the elbow flexors or the knee flexors in young female adults (Paschalis et al., 2010). If the magnitude of muscle damage is different amongst age groups, it seems reasonable to assume that proprioception changes after eccentric exercise are also different between children and adults. However, this has not been investigated in previous studies. It is interesting to examine whether eccentric exercise affects PS and JRA differently between different age groups, since impairment of proprioception potentially results in severe injury, especially during the rapid growth period (Ratel and Martin, 2015).

Some studies reported that a single bout of 40 maximal eccentric contractions of the unilateral elbow flexors (Barnes et al., 2010) or 60 high-intensity eccentric contractions of the bilateral knee extensors (Barnes et al., 2010; Lin et al., 2017) increased central arterial stiffness measured by carotid-femoral pulse-wave velocity (cfPWV), suggesting that local muscle damage could affect systemic vascular function. Niboshi et al. (2006) showed that brachial-ankle pulse-wave velocity gradually increased with growth in females (9–11 y: 919 ± 104 cm/s, 12–14 y: 932 ± 118 cm/s, 15–17 y: 952 ± 103 cm/s) and males (9–11 years: 941 ± 102 cm/s, 12–14 years: 947 ± 117 cm/s, and 15–17 years: 1041 ± 107 cm/s). Sawabe et al. (2005) demonstrated that a decrease in the elastic properties caused by the degeneration of the arterial wall resulted in an increase in aortic PWV, which began in childhood and progressed with ageing. It is not known whether increases in arterial stiffness after eccentric exercise are different, and the relationship between muscle damage and artery stiffness changes after eccentric exercise is also different between adults and children.

No previous study has compared pre-pubescent, pubescent and post-pubescent for changes in muscle damage markers, proprioception and arterial stiffness measures in a single study. To safely and effectively prescribe eccentric exercise to children, it is necessary to understand their responses to a typical eccentric exercise, especially in girls, because of the lack of information about muscle damage in pre-pubescent and pubescent females. Chen et al. (2014) showed that changes in muscle damage markers were significantly attenuated after the second bout of the same exercise, and the magnitude of the reduction in the muscle markers (i.e., the repeated bout effect) was similar among pre-adolescent, adolescent and post-adolescent males. However, no previous study has investigated the repeated bout effect on muscle damage, proprioception and arterial stiffness in pre-adolescent, adolescent and post-adolescent females.

Therefore, the aim of the present study was to compare changes in several indirect markers of muscle damage, proprioception and arterial stiffness following the first and second bouts of eccentric contractions of the elbow flexors between pre-pubescent, pubescent and post-pubescent females. It was hypothesized that the magnitude of the changes in muscle damage markers, proprioception and arterial stiffness after the exercise would be smaller in children than adults, and the changes would become greater with maturation, but the magnitude of the repeated bout effect would be similar between the groups.

## Materials and methods

### Participants

A group of pre-pubescent (9–10 y, *n* = 13), pubescent (14–15 y, *n* = 13) and post-pubescent females (20–24 y, *n* = 13), who had not performed any regular resistance training in the past 1 year, participated in this study. The sample size was estimated using the data from our previous study (Chen et al., 2014) in which pre-pubescent (9–10 y), pubescent (14–15 y) and post-pubescent males (20–25 y) were compared for muscle damage after the first and second bouts of maximal eccentric exercise of the elbow flexors. Based on the effect size of 1 for a possible difference in muscle strength changes after eccentric exercise between pre-pubescent and post-pubescent groups, with the alpha level of 0.05 and power (1–β) of 0.80, it was estimated that at least 9 participants per group were necessary by G^*^Power (G^*^Power 3.1.9.2, Heinrich-Heine-Universitat Dusseldorf, Dusseldorf, Germany; http://www.gpower.hhu.de/).

The participants signed the informed consent document before participating in this study that had been approved by a local Institutional Ethics Review Board. For the pre-pubescent and pubescent girls, their guardians also read the informed consent document and signed. The study was conducted in conformity with the policy statement regarding the use of human subjects by the Declaration of Helsinki. The participants were screened before their participation in this study to confirm that they had no neuromuscular diseases and musculoskeletal problems for the upper extremities. They were asked and reminded to refrain from unaccustomed exercise or vigorous physical activity, maintain their normal dietary and sleep habits, and not to take any anti-inflammatory drugs or nutritional supplements during the whole experimental period including the 2 weeks between the two eccentric exercise bouts. We regularly reminded the participants to follow these instructions, but these were not recorded.

To investigate the influence of maturation on changes in muscle damage, proprioception, and arterial stiffness markers after the initial and secondary bouts of eccentric exercise of the elbow flexors, the present study chose the three different age groups (9–10, 14–15, and ≥20 y) in the same way to our previous study (Chen et al., 2014), in the assumption that each age group would be classified into the stage of pre-pubescent, pubescent, and post-pubescent, respectively. To confirm this, their bone age was assessed by an experienced orthopedist using radiographs of the left hand bone according to the Greulich and Pyle age development scales (Greulich and Pyle, 1959). Some studies used peak height velocity as an indicator of maturation status (Little et al., 2000; Mirwald et al., 2002; Philipaerts et al., 2006), but the present study did not use it, because the equations of peak height velocity established by Mirwald et al. (2002) were only validated for girls aged between 9 and 13 years. Their mean (± SD) bone age for the pre-pubescent, pubescent and post-pubescent groups was 9.9 ± 0.3 y, 14.9 ± 0.3 y, and ≥18 y, respectively. It should be noted that the bone age was similar to the chronological age as shown in Table 1. Serum estrogen concentration was lower for the pre-pubescent group (19.8 ± 4.7 pg/mL) when compared with the pubescent (115.2 ± 39.9 pg/mL) and post-pubescent groups (102.5 ± 32.6 pg/mL), without a significant difference between the latter two groups. Their height, body mass, maximal voluntary isometric contraction strength at an elbow angle of 90°, maximal voluntary isokinetic (60°s^−1^) concentric contraction torque of the elbow flexors and extensors, and muscle passive stiffness were also significantly different among the groups. However, no significant difference in the range of motion of the elbow joint was found.

**Table 1:** Physiological characteristics (age, height, body mass, body mass index: BMI, left-hand bone age), resting serum estrogen concentration and baseline values of dependent variables; maximal voluntary isokinetic concentric contraction torque of the elbow flexors (MVC-EF) and extensors (MVC-EE), range of motion (ROM), and muscle passive stiffness (MPS), plasma creatine kinase (CK) activity and myoglobin (Mb) concentration, position sense (PS) and joint reaction angle (JRA) at 45° of elbow flexion, resting pulse-wave velocity measured between carotid and femoral artery (cfPWV) for pre-pubescent, pubescent and post-pubescent groups.

	**Pre-pubescent**	**Pubescent**	**Post-pubescent**	**F and P values**
Age (y)	10.3 ± 0.7^*^^#^ (9.9–10.7)	14.4 ± 0.5^*^ (14.1–14.7)	21.3 ± 1.3 (20.1–22.9)	*F*_(2, 24)_ = 461.97 *P <* 0.001
Height (cm)	141.7 ± 6.4^*^^#^ (138.0–145.4)	155.3 ± 5.3^*^ (152.3–158.4)	160.5 ± 4.5 (157.9–163.1)	*F*_(2, 24)_ = 35.47 *P <* 0.001
Body mass (kg)	40.4 ± 4.6^*^^#^ (37.7–43.0)	48.3 ± 3.8^*^ (46.1–50.5)	55.4 ± 6.7 (51.6–59.3)	*F*_(2, 24)_ = 30.02 *P* < 0.001
BMI (kg/m^2^)	20.2 ± 2.6 (18.7–21.6)	20.1 ± 1.4 (19.2–20.9)	21.5 ± 2.4 (20.1–22.9)	*F*_(2, 24)_ = 1.99 *P* = 0.16
Bone age (y)	9.9 ± 0.3^*^^#^ (9.8–10.1)	14.9 ± 0.3^*^ (14.8–15.1)	≥18.0 (18.0–18.0)	*F*_(2, 24)_ = 4044.00 *P* < 0.001
Estrogen (pg/mL)	19.8 ± 4.7^*^^#^ (17.1–22.6)	115.2 ± 39.9 (93.0–137.3)	102.5 ± 32.6 (83.6–121.3)	*F*_(2, 24)_ = 44.94 *P* < 0.001
MVIC-EF (kg)	9.8 ± 1.0^*^^#^ (9.1–10.5)	12.2 ± 1.2^*^ (11.1–13.2)	17.2 ± 2.8 (15.0–19.4)	*F*_(2, 24)_ = 58.29 *P* < 0.001
MVC-EF (Nm)	10.0 ± 0.9^*^^#^ (9.4–10.5)	14.3 ± 1.1^*^ (13.7–14.9)	19.1 ± 1.4 (18.3–19.9)	*F*_(2, 24)_ = 204.87 *P* < 0.001
MVC-EE (Nm)	13.7 ± 1.5^*^^#^ (12.8–14.5)	18.1 ± 2.1^*^ (16.9–19.3)	22.9 ± 3.6 (20.8–24.9)	*F*_(2, 24)_ = 46.14 *P* < 0.001
ROM (^O^)	145.1 ± 3.8 (142.9–147.3)	145.4 ± 5.3 (142.3–148.4)	146.5 ± 5.6 (143.3–149.7)	*F*_(2, 24)_ = 0.43 *P* = 0.66
MPS (mm/kg)	15.9 ± 1.9^*^^#^ (15.3–16.5)	14.4 ± 2.2 (13.6–15.1)	13.7 ± 1.7 (13.3–14.1)	*F*_(2, 24)_ = 4.86 *P* = 0.016
CK (IU/L)	72.7 ± 3.5^*^ (70.7–74.7)	81.4 ± 4.6 (78.8–84.1)	87.4 ± 4.5 (84.8–89.9)	*F*_(2, 24)_ = 36.40 *P* < 0.001
Mb (μg/L)	21.0 ± 0.0 (21.0–21.0)	21.1 ± 0.3 (20.9–21.2)	21.0 ± 0.0 (21.0–21.0)	*F*_(2, 24)_ = 1.00 *P* = 0.38
PS (^O^)	41.8 ± 1.9^*^ (40.7–42.8)	42.8 ± 2.2^*^ (41.6–44.0)	46.0 ± 2.7 (44.6–47.6)	*F*_(2, 24)_ = 10.69 *P* < 0.001
JRA (^O^)	38.4 ± 1.4^*^ (37.6–39.3)	39.7 ± 1.6^*^ (38.8–40.6)	41.3 ± 1.7 (40.4–42.3)	*F*_(2, 24)_ = 10.51 *P* < 0.001
cfPWV (cm/s)	902.8 ± 59.8^*^^#^ (866.7–939.0)	967.0 ± 61.4^*^ (929.9–1004.9)	1102.7 ± 73.1 (1058.8–1146.8)	*F*_(2, 24)_ = 4.97 *P* = 0.016

*Mean ± SD, 95% confidence intervals (in brackets), and F- and P-values by one-way ANOVA for the comparison between the three groups are shown for each variable*.

* *Significantly (P < 0.05) different from the post-pubescent group*,

# *Significantly (P < 0.05) different from the pubescent group*.

### Experimental protocol

All participants performed two bouts of eccentric exercise of the elbow flexors (EC1, EC2) with non-dominant arm separated by 2 weeks based on the previous studies showing the repeated bout effect for this time interval for the elbow flexors (Howatson and van Someren, 2007; Starbuck and Eston, 2012; Chen et al., 2016). A familiarization session was held 3 days prior to EC1, in which the participants experienced the measurements of upper arm circumference, muscle soreness, range of motion, maximal concentric torque of the elbow flexors and extensors, and maximal isometric strength in this order. The investigator demonstrated the maximal eccentric exercise of the elbow flexors but the participants did not perform any eccentric contraction, since it has been reported that only a few maximal eccentric contractions could confer some protective effect (Nosaka et al., 2001).

Dependent variables consisted of maximal concentric torque of the elbow flexors and extensors, range of motion, plasma CK activity and myoglobin concentration, muscle passive stiffness, muscle soreness, position sense (PS), joint reaction angle (JRA), and pulse-wave velocity measured between carotid and femoral artery (cfPWV). The measurements were taken before, immediately after, and 1, 2, 3, 4, and 5 days post-exercise for all variables except for muscle soreness, plasma CK activity and myoglobin concentration. The muscle soreness was assessed at all time points except immediately post-exercise, and CK and myoglobin were measured immediately before, and 2 and 4 days after exercise. Reliability of each dependent variable measurement was determined by an intraclass correlation coefficient (ICC) and coefficient of variation (CV) using the values taken at 2 days and immediately before EC1 for all participants. The ICC and CV (shown in parentheses) values for maximal concentric torque of the elbow flexors and extensors, range of motion, plasma CK activity and myoglobin concentration, PS, JRA, and cfPWV ranged between 0.99 (7.5%) in muscle passive stiffness to 0.85 (7.0%) in CK for the pre-pubescent, between 0.98 (8.0%) in maximal concentric torque of the elbow flexors to 0.92 (5.9%) in cfPWV for the pubescent, and between 0.97 (3.8%) in ROM) to 0.88 (6.4%) in cfPWV for the post-pubescent group. Since no muscle soreness was detected at baseline from any of the participants, the CV and ICC values of muscle soreness measure were not established in the present study. However, the reliability of the muscle soreness assessment has been established in our previous studies (Chen et al., 2014, 2016, 2017a). Changes in the dependent variables after EC1 and EC2 were compared among the three age groups.

### Eccentric exercise

To determine a dumbbell weight for the eccentric exercise, each participant stood up with the non-dominant arm relaxed and hang down by the side, with the back of participant's body being touched to a wall. Maximal isometric strength was measured at 90° elbow flexion with 0° shoulder flexion using a loadcell (Model DFG51; Omega Engineering, Stamford, CT) connected to a digital recorder (Biopac Systems, Goleta, CA) with a gravity correction. Each participant wore a cuff in the wrist of the non-dominant arm, and the cuff was connected by an adjustable cable to the loadcell that was fixed on the floor. The participants were well familiarized with the protocol in the familiarization session and before the baseline measure. They were instructed to generate maximal force for 3 s at 90° elbow flexion for 3 times with a 45-s rest between trials, and strong verbal encouragement was provided in a consistent manner during all tests. If the values of the three measurements were more than 10% different, 1–3 measurements were added to make sure the reliability. We assumed that each participant did her best to generate maximal force, and considered that the values represented maximal capacity. The highest value of the three trials (or more than three trials) was used to determine the dumbbell weight corresponding to 60% the maximal isometric strength. This load was found to induce muscle damage, based on our pilot study.

In the exercise, a dumbbell weight was set at 60% of individual's maximal isometric strength as mentioned above, and the participants were asked to lower the dumbbell slowly from an elbow flexed (90°) to an elbow fully extended position (0°) in 5 s, while the investigator counted 0, 1, 2, 3, 4, and 5 to indicate the movement. The exercise consisted of 5 sets of 6 eccentric contractions with 15 s between contractions and 2 min between sets. After each eccentric contraction, the investigator removed the dumbbell, and each participant returned the arm to the starting position without the dumbbell. All participants were able to complete the exercise bouts without spotting.

### Dependent variables

#### Maximal concentric strength of the elbow flexors and extensors

Maximal concentric strength of the elbow flexors and extensors were measured at the angular velocity of 60°·s^−1^ for the range of motion of 140°. Three continuous maximal voluntary concentric contractions were performed in both directions (Chen et al., 2016, 2017a; Tseng et al., 2016). The torque, position (joint angle) and displacement signals of each contraction were recorded to a computer connected to the isokinetic dynamometer. The raw data were filtered and smoothed, and peak torque was analyzed by the software of the Biodex Medical Systems, and the highest value of the three trials was used for subsequent analysis (Wang et al., 2014; Chen et al., 2015; Shih et al., 2016).

#### Range of motion (ROM)

ROM was determined as the difference in the joint angles between maximal flexion (FANG) and extension (EANG) of elbow joint (Chen et al., 2016). The FANG was measured when the participant maximally flexed the elbow joint while keeping the elbow joint at the side of the body in a standing position. The EANG was measured when the participant attempted to extend the elbow joint maximally with the elbow held by the side. A plastic goniometer was used for the measures, and three measurements were taken for each angle, and the average of the three measurements was used to calculate range of motion by subtracting FANG from EANG (Chen et al., 2014).

#### Muscle passive stiffness

A myotonometer® (Neurogenic Technologies, Inc, Missoula, MT, USA) has been shown to be valid for assessing muscle passive stiffness of a relaxed muscle (Leonard et al., 2004; Hung et al., 2010). Each participant sat on a chair with the forearm being relaxed and placed on a padded table at the shoulder angle of 80° and the elbow joint angle of 10° flexion. The head of the myotonometer probe was placed along the longitudinal axis of the biceps brachii muscle at 50% of the distance from the acromion process of the clavicle to the lateral epicondyle of the humerus. The probe pressure was increased automatically from 0.25 to 2.0 kg every 0.25 kg, and this was repeated 3 times according to the standardized method used in the previous study (Hung et al., 2010). The software to operate the myotonometer recorded tissue displacement in response to each pressure, and the relationship between the pressure and displacement was obtained, providing a force-displacement curve, and the area under the curve (AUC) was computed. The AUC between 0.25 and 2.00 kg was used as an indicator of muscle passive stiffness (Chen et al., 2014).

#### Muscle soreness

The level of muscle soreness was quantified using a visual analog scale that had a 100-mm continuous line with “not sore at all” on one side (0 mm) and “very, very sore” on the other side (100 mm) based on the previous study (Chen et al., 2014, 2017b; Tseng et al., 2016). The investigator asked the participant to rate her perceived soreness on the visual analog scale when the elbow joint was passively extended and flexed for the range of motion that used for the maximal concentric strength measures (Chen et al., 2014).

#### Plasma CK activity and myoglobin concentration

Approximately 3-ml of venous blood was withdrawn by a standard venipuncture technique from the cubital fossa region of the arm, and centrifuged for 10 min to extract plasma, which was stored at −80°C until analyses. Plasma CK activity was assayed spectrophotometrically by an automated clinical chemistry analyzer (Model 7080, Hitachi, Co. Ltd., Tokyo, Japan) using a commercial test kit (Roche Diagnostics, Indianapolis, IN, USA). Plasma myoglobin concentration was measured by an automated clinical chemistry analyzer (Model Elecsys 2010, F. Hoffmann-La Roche Ltd., Tokyo, Japan) using a commercial test kit (Roche Diagnostics, Indianapolis, IN, USA). Each sample was analyzed in duplicate, and the average value of two measures was used for subsequent statistical analysis.

#### Position sense (PS) and joint reaction angle to release (JRA) of the elbow flexors

PS and JRA measures were adopted from the previous study (Paschalis et al., 2010), and the details of the measurements can be found in the article. Briefly, each participant seated and blindfolded for both PS and JRA testing, as the same position required as MVC testing on the isokinetic dynamometer. As for PS, the investigator positioned the testing arm of each participant to one of the three angles (30°, 45°, 60°) in random order, held it for 10 s, and returned it passively to a full elbow extension (0°), then asked the participant to stop the elbow flexion movement at 20°·s^−1^ by pressing a stop button by the hand of the opposite arm at the perceived target angle. For PS test, the testing arm of the participant was passively moved by the investigator from 90° elbow flexion to a target angle (e.g., 45°) slowly (approximately 20°·s^−1^), then the testing arm was stopped and fully relaxed at a target angle for 5–10 s when it reached the target angle showing on the computer screen, then the investigator suddenly released the lever arm without warning. The participant was asked to stop the lever arm as soon as the investigator released the lever arm. The difference between the target angle and the actual angle (PS) or the angle at stopping (JRA) was recorded, respectively. The time between trials for the same test was 10 s, and 2 min of rest were inserted between different angles for both PS and JRA. The difference from the target angle of the mean value of the two closest to the reference angle among four trials was used for further analysis.

#### Arterial stiffness

Each participant rested in the supine position at least for 10 min before this measurement. Arterial stiffness was assessed by carotid-femoral pulse wave velocity (cfPWV) that calculated from the traveling distance and foot-to-foot wave transit time between two arterial recording sites (Huang et al., 2015, 2016). Non-invasive pulse tonometer (SPT-301, Millar Inc., Houston, TX, USA) that was connected to a physiological signaling processing system (MP36, Biopac, Goleta, CA, USA) was used to detect pulse waves on the carotid and femoral arteries, and cfPWV (cm/s) was calculated by the distance (cm) and pulse waves (s) between carotid and femoral arteries (Huang et al., 2015, 2016).

### Statistical analyses

Data were assessed by a Shapiro–Wilk test for the normality and a Levene test for the homogeneity of variance assumption. Baseline values of the criterion measures were compared amongst groups by a one-way analysis of variance (ANOVA). There were two main comparisons in the study; (1) comparison between the pre-pubescent, pubescent and post-pubescent groups for the changes in each dependent variable after the first bout of eccentric exercise, and (2) comparison between the first and second bouts for each group separately for the repeated bout effect. For these, a three-way ANOVA (group × bout × time) was performed first to identify whether a significant interaction effect existed for changes in each variable before and after the first and second bouts of eccentric exercise. If a significant interaction effect was found, a series of a mixed-design two-way ANOVAs were performed to compare two groups (i.e., pre-pubescent vs. pubescent, pre-pubescent vs. post-pubescent, pubescent vs. post-pubescent), and to compare between the first and second exercise bouts for each group separately, followed by a Tukey's *post-hoc* test when a significant interaction effect was found, as required. The effect size of the ANOVA results was shown by an eta-squared (η^2^) where necessary.

An index of protective effect conferred by EC1 against EC2 was assessed using the values at 1 day post-exercise for maximal concentric strength of the elbow flexors and range of motion, and the highest post-exercise values for muscle passive stiffness, muscle soreness, plasma CK activity and myoglobin concentration as well as cfPWV, and lowest post-exercise values for PS and JRA using the following equation of the protection index; (EC1–EC2)/EC1 × 100 (Chen et al., 2014). The index for each measure was compared among the groups by a one-way ANOVA, and a Tukey's *post-hoc* test was performed when a significant main effect was found. By comparing the values of EC1 and EC2 used for the protection index, effect size for the difference between EC1 and EC2 for each variable was calculated by Cohen's *d*, and was considered 0.2, 0.5, and 0.8 as a small, medium, and large effect, respectively (Cohen, 1988). Statistical significance was set at *P* ≤ 0.05. Data are presented as mean ± SD, unless otherwise stated. Statistical analyses were performed using the SAS 9.4 software (https://www.sas.com/en_us/software/sas9.html).

## Results

### Baseline measures

No significant differences in the baseline measures of any dependent variables were observed between EC1 and EC2 for each group. As shown in Table 1, maximal concentric strength and cfPWV were significantly different across groups, and post-hoc test showed that pre-pubescent < pubescent < post-pubescent. Muscle passive stiffness was significantly smaller for the pre-pubescent than other two groups. Position sense and reaction angle to release, and plasma CK activity were significantly smaller for the pre-pubescent and pubescent groups than the post-pubescent group.

### Muscle damage markers

**Figure 1:**
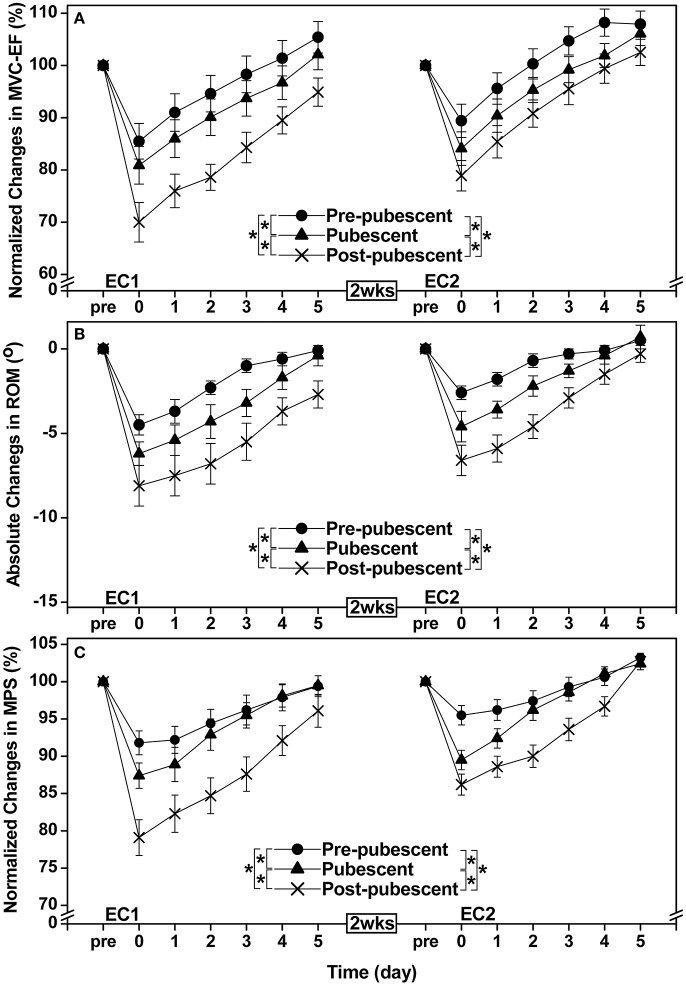
Normalized changes in maximal voluntary concentric contraction torque of the elbow flexors (MVC-EF, **A**), absolute changes in range of motion of the elbow joint (ROM, **B**), and normalized changes in muscle passive stiffness (MPS, **C**) from baseline (pre) to immediately (post) and 1–5 days after the first (EC1) and second (EC2) eccentric exercise bouts for the pre-pubescent, pubescent and post-pubescent groups. A significant (*P* < 0.05) interaction (group × time) effect based on a pairwise comparison between two groups by a mixed design two-way ANOVA is shown by ^*^ in the figure. *P, F*, and eta-squared (η^2^) values shown by a mixed design two-way ANOVA for the comparison between two groups are shown below. Pre-pubescent vs. Pubescent. EC1: MVC-EF *F*_(5, 60)_ = 5.91, *P* < 0.001, η^2^ = 0.313. ROM *F*_(5, 60)_ = 3.30, *P* = 0.006, η^2^ = 0.203. MPS *F*_(5, 60)_ = 2.27, *P* = 0.045, η^2^ = 0.149. EC2: MVC-EF *F*_(5, 60)_ = 7.93, *P* < 0.001, η^2^ = 0.379. ROM *F*_(5, 60)_ = 2.75, *P* = 0.018, η^2^ = 0.174. MPS *F*_(5, 60)_ = 8.72, *P* < 0.001, η^2^ = 0.402. Pre-pubescent vs. Post-pubescent. EC1: MVC-EF *F*_(5, 60)_ = 36.75, *P* < 0.001, η^2^ = 0.739. ROM *F*_(5, 60)_ = 12.28, *P* < 0.001, η^2^ = 0.486. MPS *F*_(5, 60)_ = 17.22, *P* < 0.001, η^2^ = 0.570. EC2: MVC-EF *F*_(5, 60)_ = 17.04, *P* < 0.001, η^2^ = 0.567. ROM *F*_(5, 60)_ = 6.56, *P* < 0.001, η^2^ = 0.335. MPS *F*_(5, 60)_ = 15.03, *P* < 0.001, η^2^ = 0.536. Pubescent vs. Post-pubescent; EC1: MVC-EF *F*_(5, 60)_ = 13.61, *P* < 0.001, η^2^ = 0.511. ROM *F*_(5, 60)_ = 2.91, *P* = 0.013, η^2^ = 0.183. MPS *F*_(5, 60)_ = 4.46, *P* = 0.001, η^2^ = 0.255. EC2: MVC-EF *F*_(5, 60)_ = 4.78, *P* < 0.001, η^2^ = 0.269. ROM *F*_(5, 60)_ = 3.07, *P* = 0.010, η^2^ = 0.191. MPS *F*_(5, 60)_ = 5.42, *P* < 0.001, η^2^ = 0.294. P, F and eta-squared (η^2^) values of a pairwise comparison between bouts for each group by a mixed design two-way ANOVA are shown below. Pre-pubescent: MVC-EF *F*_(5, 60)_ = 3.29, *P* = 0.006, η^2^ = 0.202. ROM *F*_(5, 60)_ = 5.80, *P* < 0.001, η^2^ = 0.308. MPS *F*_(5, 60)_ = 5.19, *P* < 0.001, η^2^ = 0.285. Pubescent: MVC-EF *F*_(5, 60)_ = 3.60, *P* = 0.003, η^2^ = 0.21. ROM *F*_(5, 60)_ = 12.18, *P* < 0.001, η^2^ = 0.484. MPS *F*_(5, 60)_ = 3.19, *P* = 0.008, η^2^ = 0.197. Post-pubescent: MVC-EF *F*_(5, 60)_ = 8.72, *P* < 0.001, η^2^ = 0.401. ROM *F*_(5, 60)_ = 6.65, *P* < 0.001, η^2^ = 0.338. MPS *F*_(5, 60)_ = 2.93, *P* = 0.012, η^2^ = 0.184.

**Figure 2:**
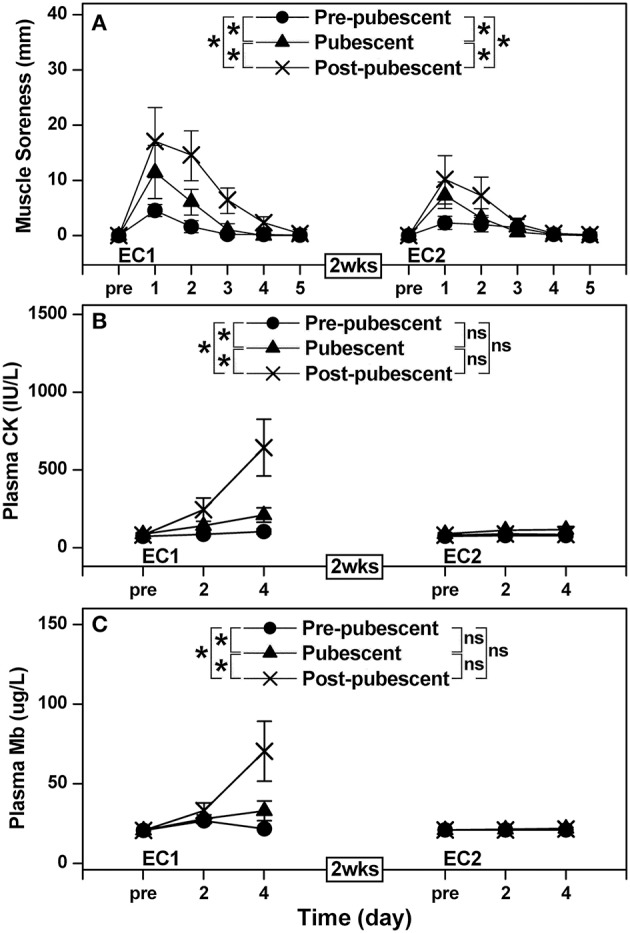
Changes in muscle soreness **(A)**, and changes in plasma creatine kinase activity (CK, **B**) and myoglobin concentration (Mb, **C**) before, and 1–5 days or 2 and 4 days following the first (EC1) and second (EC2) eccentric exercise bout for the pre-pubescent, pubescent and post-pubescent groups. A significant (*P* < 0.05) interaction (group × time) effect based on a pairwise comparison between two groups by a mixed design two-way ANOVA is shown by ^*^ in the figure. *P, F*, and eta-squared (η^2^) values shown by a mixed design two-way ANOVA for the comparison between two groups are shown below. Pre-pubescent vs. Pubescent; EC1: muscle soreness *F*_4, 48_ = 6.04, *P* < 0.001, η^2^ = 0.317. CK *F*_(2, 24)_ = 4.99, *P* = 0.015, η^2^ = 0.277. Mb *F*_(2, 24)_ = 5.63, *P* = 0.009, η^2^ = 0.302. Pre-pubescent vs. Pubescent; EC2: muscle soreness *F*_(4, 48)_ = 16.92, *P* < 0.001, η^2^ = 0.565. CK *F*_(2, 24)_ = 2.49, *P* = 0.103, η^2^ = 0.160. Mb *F*_(2, 24)_ = 1.31, *P* = 0.288, η^2^ = 0.091. Pre-pubescent vs. Post-pubescent; EC1: muscle soreness *F*_(4, 48)_ = 4.57, *P* = 0.001, η^2^ = 0.260. CK *F*_(2, 24)_ = 9.74, *P* = 0.001, η^2^ = 0.428. Mb *F*_(2, 24)_ = 5.19, *P* = 0.013, η^2^ = 0.285. Pre-pubescent vs. Post-pubescent; EC2: muscle soreness *F*_(4, 48)_ = 12.01, *P* < 0.001, η^2^ = 0.480. CK *F*_(2, 24)_ = 2.79, *P* = 0.080, η^2^ = 0.177. Mb *F*_(2, 24)_ = 2.24, *P* = 0.126, η^2^ = 0.147. Pubescent vs. Post-pubescent; EC1: muscle soreness *F*_(4, 48)_ = 2.94, *P* = 0.019, η^2^ = 0.184. CK *F*_(2, 24)_ = 8.76, *P* = 0.001, η^2^ = 0.403. Mb *F*_(2, 24)_ = 4.67, *P* = 0.019, η^2^ = 0.264. Pubescent vs. Post-pubescent; EC2: muscle soreness *F*_(4, 48)_ = 2.39, *P* = 0.047, η^2^ = 0.155. CK *F*_(2, 24)_ = 0.88, *P* = 0.429, η^2^ = 0.063. Mb *F*_(2, 24)_ = 0.68, *P* = 0.515, η^2^ = 0.050. P, F and eta-squared (η^2^) values of a pairwise comparison between bouts for each group by a mixed design two-way ANOVA are shown below. Pre-pubescent; EC1 vs. EC2: muscle soreness *F*_(4, 48)_ = 8.33, *P* < 0.001, η^2^ = 0.410. CK *F*_(2, 24)_ = 2.25, *P* = 0.126, η^2^ = 0.147. Mb *F*_(2, 24)_ = 2.00, *P* = 0.156, η^2^ = 0.133. Pubescent; EC1 vs. EC2: muscle soreness *F*_(4, 48)_ = 13.20, *P* < 0.001, η^2^ = 0.504. CK *F*_(2, 24)_ = 10.00, *P* = 0.001, η^2^ = 0.435. Mb *F*_(2, 24)_ = 5.77, *P* = 0.008, η^2^ = 0.307. Post-pubescent; EC1 vs. EC2: muscle soreness *F*_(4, 48)_ = 7.30, *P* < 0.001, η^2^ = 0.360. CK *F*_(2, 24)_ = 11.33, *P* < 0.001, η^2^ = 0.466. Mb *F*_(2, 24)_ = 5.19, *P* = 0.003, η^2^ = 0.285.

All criterion measures except maximal concentric strength of the elbow extensors changed significantly (*P* < 0.05) following EC1 for all groups. A significant group × time interaction effect was evident for the changes in maximal concentric strength of the elbow flexors [*F*_(10, 120)_ = 6.99, *P* < 0.001, η^2^ = 0.350], range of motion [*F*_(10, 120)_ = 2.01, *P* = 0.037, η^2^ = 0.134], muscle passive stiffness [*F*_(10, 120)_ = 2.71, *P* = 0.005, η^2^ = 0.173], muscle soreness [*F*_(8, 96)_ = 6.14, *P* < 0.001, η^2^ = 0.338], plasma CK activity [*F*_(4, 48)_ = 7.68, *P* < 0.001, η^2^ = 0.390] and plasma Mb concentration [*F*_(4, 48)_ = 4.41, *P* = 0.004, η^2^ = 0.269] after EC1. When comparing two groups, the magnitude of the changes was significantly smaller for the pre-pubescent and pubescent groups when compared with the post-pubescent group, and the changes were smaller (*P* < 0.05) for the pre-pubescent than pubescent group (Figures 1, 2).

After EC2, maximal concentric strength of the elbow flexors, range of motion, muscle passive stiffness, muscle soreness, and plasma CK activity changed significantly (*P* < 0.05), although the magnitude of changes was smaller than that after EC1. A significant group × time interaction effect was evident for the changes in maximal concentric strength of the elbow flexors [*F*_(10, 120)_ = 3.78, *P* < 0.001, η^2^ = 0.225], range of motion [*F*_(10, 120)_ = 3.16, *P* = 0.001, η^2^ = 0.197], muscle passive stiffness [*F*_(10, 120)_ = 8.56, *P* < 0.001, η^2^ = 0.397] and muscle soreness [*F*_(8, 96)_ = 7.03, *P* < 0.001, η^2^ = 0.370] after EC2. When comparing two groups, the changes were smaller (*P* < 0.05) for the pre-pubescent and pubescent groups than the post-pubescent group for all criterion measures except plasma CK activity and plasma Mb concentration. Changes in maximal concentric strength of the elbow flexors, range of motion, muscle passive stiffness, and muscle soreness were smaller (*P* < 0.05) for the pre-pubescent than the pubescent group (Figures 1, 2).

When comparing between EC1 and EC2, changes in all criterion measures were smaller (*P* < 0.05) following EC2 than EC1 for the pubescent and post-pubescent groups, but no significant differences between bouts were found for CK (*P* > 0.05) and myoglobin (*P* > 0.05) in the pre-pubescent group (Figures 1, 2).

### Proprioception

**Figure 3:**
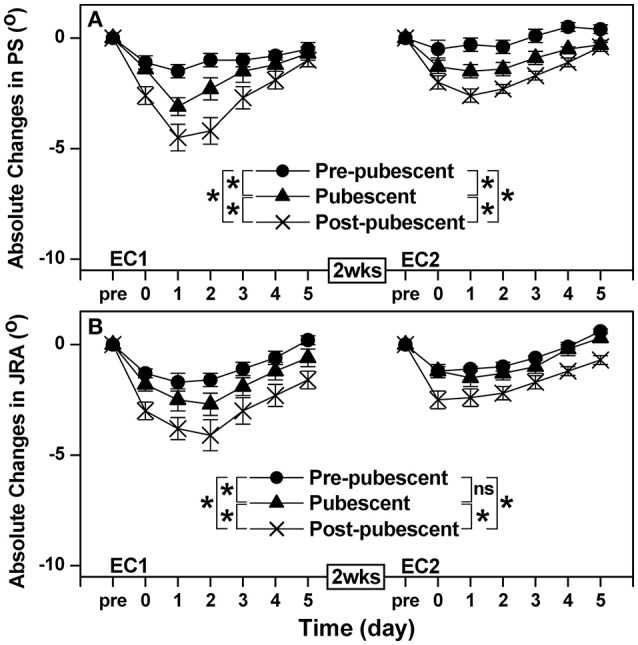
Absolute changes in angle of position sense (PS, **A**) and joint reaction angle (JRA, **B**) from the baseline (Pre, 0), immediately after exercise (0) and 1–5 days following the first (EC1) and second (EC2) eccentric exercise bout for the pre-pubescent, pubescent and post-pubescent groups. A significant (*P* < 0.05) interaction (group × time) effect based on a pairwise comparison between two groups by a mixed design two-way ANOVA is shown by ^*^ in the figure. *P, F*, and eta-squared (η^2^) values shown by a mixed design two-way ANOVA for the comparison between two groups are shown below. Pre-pubescent vs. Pubescent; EC1: PS *F*_(5, 60)_ = 6.69, *P* < 0.001, η^2^ = 0.340. JRA *F*_(5, 60)_ = 3.08, *P* = 0.009, η^2^ = 0.191. Pre-pubescent vs. Pubescent; EC2: PS *F*_(5, 60)_ = 6.71, *P* < 0.001, η^2^ = 0.341. JRA *F*_(5, 60)_ = 0.54, *P* = 0.776, η^2^ = 0.040. Pre-pubescent vs. Post-pubescent; EC1: PS *F*_(5, 60)_ = 14.03, *P* < 0.001, η^2^ = 0.519. JRA *F*_(5, 60)_ = 11.96, *P* < 0.001, η^2^ = 0.479. Pre-pubescent vs. Post-pubescent; EC2: PS *F*_(5, 60)_ = 20.30, *P* < 0.001, η^2^ = 0.610. JRA *F*_(5, 60)_ = 3.41, *P* = 0.005, η^2^ = 0.208. Pubescent vs. Post-pubescent; EC1: PS *F*_(5, 60)_ = 2.65, *P* = 0.031, η^2^ = 0.169. JRA *F*_(5, 60)_ = 6.98, *P* < 0.001, η^2^ = 0.349. Pubescent vs. Post-pubescent; EC2: PS *F*_(5, 60)_ = 2.86, *P* = 0.014, η^2^ = 0.180. JRA *F*_(5, 60)_ = 4.66, *P* < 0.001, η^2^ = 0.264. P, F and eta-squared (η_p_2) values of a pairwise comparison between bouts for each group by a mixed design two-way ANOVA are shown below. Pre-pubescent; EC1 vs. EC2: PS *F*_(5, 60)_ = 5.29, *P* < 0.001, η^2^ = 0.274. JRA *F*_(5, 60)_ = 0.97, *P* = 0.448, η^2^ = 0.070. Pubescent; EC1 vs. EC2: PS *F*_(5, 60)_ = 4.06, *P* < 0.001, η^2^ = 0.238. JRA *F*_(5, 60)_ = 2.78, *P* = 0.017, η^2^ = 0.176. Post-pubescent; EC1 vs. EC2: PS *F*_(5, 60)_ = 5.70, *P* < 0.001, η^2^ = 0.305. JRA *F*_(5, 60)_ = 8.89, *P* < 0.001, η^2^ = 0.406.

Changes in PS and JRA of three angles testing following EC1 and EC2 showed similar pattern for each group, thus only 45° of elbow flexion in angle testing was presented in Figure 3. A significant group × time interaction effect was evident for the changes in PS [*F*_(10, 120)_ = 7.20, *P* < 0.001, η^2^ = 0.356] and JRA [*F*_(10, 120)_ = 7.50, *P* < 0.001, η^2^ = 0.366] after EC1. Changes in PS and JRA following EC1 were greater (*P* < 0.05) for the post-pubescent group than the pre-pubescent and pubescent groups, and for the pubescent than pre-pubescent group (Figure 3). Similarly to EC1, a significant group × time interaction effect was also evident for the changes in PS [*F*_(10, 120)_ = 4.35, *P* < 0.001, η^2^ = 0.251] and JRA [*F*_(10, 120)_ = 2.85, *P* = 0.001, η^2^ = 0.180] after EC2. No significant (*P* > 0.05) difference between pre-pubescent and pubescent groups was found for the changes in JRA after EC2, but a significant difference between groups was evident for the other combinations for the changes in JRA and all combinations for the changes in PS (Figure 3). When comparing between EC1 and EC2, the changes in PS and JRA were smaller (*P* < 0.05) after EC2 than EC1 for all groups with the exception of JRA for the pre-pubescent group (Figure 3).

### cfPWV

**Figure 4:**
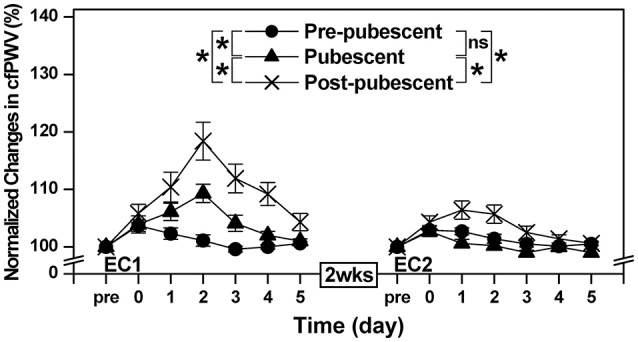
Normalized changes in carotid-femoral pulse-wave velocity (cfPWV) from baseline (Pre: 100%) immediately (post) and 1–5 days after the first (EC1) and second (EC2) eccentric exercise bouts for the pre-pubescent, pubescent and post-pubescent groups. ^*^: significant (*P* < 0.05) interaction (group × time) effect based on a pairwise comparison between groups. A significant (*P* < 0.05) interaction (group × time) effect based on a pairwise comparison between two groups by a mixed design two-way ANOVA is shown by ^*^ in the figure. *P, F*, and eta-squared (η^2^) values shown by a mixed design two-way ANOVA for the comparison between two groups are shown below. Pre-pubescent vs. Pubescent; EC1: *F*_(5, 60)_ = 8.65, *P* < 0.001, η^2^ = 0.419. Pre-pubescent vs. Pubescent; EC2: *F*_(5, 60)_ = 1.40, *P* = 0.237, η^2^ = 0.105. Pre-pubescent vs. Post-pubescent; EC1: *F*_(5, 60)_ = 41.71, *P* < 0.001, η^2^ = 0.777. Pre-pubescent vs. Post-pubescent; EC2: *F*_(5, 60)_ = 7.81, *P* < 0.001, η^2^ = 0.394. Pubescent vs. Post-pubescent; EC1: *F*_(5, 60)_ = 7.73, *P* < 0.001, η^2^ = 0.392. Pubescent vs. Post-pubescent; EC2: *F*_(5, 60)_ = 13.68, *P* < 0.001, η^2^ = 0.533. P, F and eta-squared (η^2^) values of a pairwise comparison between bouts for each group by a mixed design two-way ANOVA are shown below. Pre-pubescent; EC1 vs. EC2: *F*_(5, 60)_ = 3.18, *P* = 0.013, η^2^ = 0.209. Pubescent; EC1 vs. EC2: *F*_(5, 60)_ = 40.01, *P* < 0.001, η^2^ = 0.769. Post-pubescent; EC1 vs. EC2: *F*_(5, 60)_ = 19.24, *P* < 0.001, η^2^ = 0.616.

cfPWV increased significantly (*P* < 0.05) after EC1, and a significant group × time interaction effect was evident for the changes [*F*_(10, 120)_ = 24.01, *P* < 0.001, η^2^ = 0.667]. Increases in cfPWV following EC1 were greater (*P* < 0.05) for the post-pubescent than the pre-pubescent and pubescent groups, and the increases were smaller (*P* < 0.05) for the pre-pubescent group when compared to the pubescent group (Figure 4). After EC2, cfPWV also increased significantly (*P* < 0.05), and a significant group × time interaction effect was still evident [*F*_(10, 120)_ = 5.26, *P* < 0.001, η^2^ = 0.305]. Increases in cfPWV following EC2 were greater (*P* < 0.05) for the post-pubescent group than the pre-pubescent and pubescent groups, without a significant (*P* > 0.05) difference between the pubescent and pre-pubescent groups (Figure 4). When comparing between EC1 and EC2, the increases after EC2 were smaller (*P* < 0.05) for all groups.

### Protective effect

**Figure 5:**
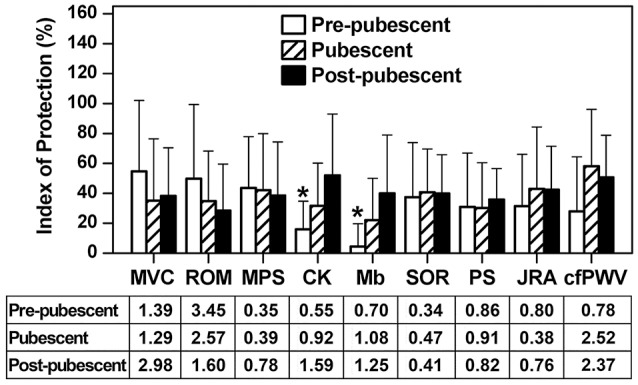
Comparison in protective index [(EC1–EC2)/EC1 × 100%] amongst the pre-pubescent, pubescent and post-pubescent groups for maximal voluntary concentric contraction torque of the elbow flexors (MVC), range of motion (ROM), muscle passive stiffness (MPS), plasma creatine kinase activity (CK), plasma myoglobin concentration (Mb) and muscle soreness (SOR), angle of position sense (PS), joint reaction angle (JRA), and carotid-femoral pulse-wave velocity (cfPWV). For the calculation of the index, the values at 1 day post-exercise were used for MVC and ROM, and highest post-exercise values were used for MPS, SOR, CK, Mb, and cfPWV, and lowest post-exercise values were used for PS and JRA. At the bottom of the figure, effect size (Cohen's *d*) for the difference in each variable between EC1 and EC2 for each group is shown. ^*^Significantly (*P* < 0.05) different from the post-pubescent group.

The magnitude of protective effect was not significantly different (*P* = 0.055–0.909) amongst the groups for any variables except plasma CK activity (*P* = 0.003) and myoglobin concentration (*P* = 0.002) as shown in Figure 5. For plasma CK activity and myoglobin concentration, the protective effect was significantly greater for post-pubescent (CK: 52 ± 41%, myoglobin: 40 ± 39%) and pubescent groups (CK: 26 ± 30%, myoglobin: 19 ± 27%) than pre-pubescent group (CK: 15 ± 17%, myoglobin: 4 ± 12%), but this was due to the smaller changes in these variables after EC1 for the pubescent and pre-pubescent groups than the post-pubescent group (Figures 2B,C). The effect size for the difference between bouts for each variable is shown for each age group (Figure 5). Most of the effect sizes were large, but the differences between the age groups were small.

## Discussion

The present study showed that changes in muscle damage, proprioception and arterial stiffness measures following the first bout of eccentric exercise of the elbow flexors were significantly smaller for the pre-pubescent and pubescent groups than post-pubescent group, and all changes except muscle passive stiffness were significantly smaller for the pre-pubescent than pubescent group (Figures 1–4). Changes in all measures after the second bout of eccentric exercise were significantly smaller than those after the initial bout for all groups (Figures 1–4), and the magnitude of the repeated bout effect for all measures except CK and myoglobin were similar among the groups (Figure 5). These results are in line with the findings of our previous study (Chen et al., 2014) showing that the magnitude of muscle damage was significantly smaller for pre-pubescent and pubescent boys than post-pubescent men, and the magnitude of the repeated bout effect was similar among the three age groups. The present study was the first to show the changes in proprioception and arterial stiffness after eccentric exercise of the elbow flexors in children.

### Muscle damage and the repeated bout effect

The present study used only indirect markers of muscle damage, thus muscle damage in histological level was not known; however, all previous studies comparing children and adults for eccentric exercise-induced muscle damage used “semi-quantitative” variables of muscle damage (Webber et al., 1989; Soares et al., 1996; Marginson et al., 2005; Gorianovas et al., 2013; Chen et al., 2014; Deli et al., 2017). As shown in Figures 1,4, changes in all markers of muscle damage after EC1 were the smallest for the pre-pubescent group, followed by the pubescent group, then the post-pubescent group. These results were similar to the findings of our previous study (Chen et al., 2014) using males who performed a maximal isokinetic eccentric exercise of the elbow flexors. The present study was the first to show that muscle damage of the elbow flexors was less in children than adults for females. In the previous study (Chen et al., 2014), an isokinetic dynamometer was used for the eccentric exercise in which the shoulder joint angle was set at 45° flexion and 0° abduction. The lesser muscle damage of children would have been due to the greater shoulder joint flexibility of children than adults, which might have affected the muscle damage in the isokinetic exercise setting in our previous study (Chen et al., 2014). This was why the present study used the exercise protocol that was thought to be less affected by the shoulder joint flexibility. In spite of the different eccentric exercise protocol, the present study still found less muscle damage in children than adults. Previous studies (Webber et al., 1989; Soares et al., 1996; Marginson et al., 2005; Gorianovas et al., 2013; Chen et al., 2014; Deli et al., 2017) also showed that children were less susceptible to eccentric exercise-induced muscle damage than adults. The present study added further evidence to support this notion.

It has been reported that age is a factor affecting the magnitude of eccentric exercise-induced muscle damage from pre-pubescent to post-pubescent males (Chen et al., 2014). We used bone age rather than somatic maturation to assess maturation of female participants, and did not use the peak height velocity, since the equations of Mirwald et al. (2002), which are most used currently, were validated only for girls aged between 9 and 13 years old. As shown in Table 1, there were distinct differences among the three age groups for bone age. It should be noted that the chronological age and bone age were similar in each age group in the present study (Table 1). The differences in resting serum estrogen concentrations were greater between the pre-pubescent and pubescent groups than between the pubescent and post-pubescent groups (Table 1). It should be noted that the present study did not standardize the stage of the menstrual cycle among the pubescent and post-pubescent females. Nevertheless, the differences in the changes in muscle damage parameters (Figures 1–2) were greater between the pubescent and post-pubescent groups than between the pre-pubescent and pubescent groups. Thus, it does not appear that the differences in serum estrogen concentration between groups can explain the clear differences in the magnitude of muscle damage among the three groups.

Differences in muscle fiber type (less fast-twitch muscle fibers in children than adults) and compliance of muscle-tendon complex (greater compliance in children than adults) may be possible factors explaining why muscle damage was smallest for the pre-pubescent group (Lexell et al., 1992; Lambertz et al., 2003; Chen et al., 2014). It has been shown that muscle damage induced by eccentric contractions occurs predominantly to fast-twitch fibers (Fridèn et al., 1983). Thus, if children have relatively fewer fast-twitch fibers, they may be less susceptible to eccentric exercise-induced muscle damage. Furthermore, musculo–tendinous stiffness is lower in children than adults (Lambertz et al., 2003), thus compliant tendon in children may act as a “buffer” to reduce mechanical strain on muscle fascicles and fibers. In the present study, muscle passive stiffness was assessed by a myotonometer, and found that biceps brachii was less stiff for the pre-pubescent than other groups. It has been reported that stiff muscles are more susceptible to eccentric exercise-induced muscle damage (McHugh et al., 1999; Chen et al., 2011). The smaller muscle passive stiffness of pre-pubescent group than other groups may contribute to less muscle damage found in the pre-pubescent group; however, this does not explain the difference in the muscle damage between the pubescent and post-pubescent groups. Further research is required to understand why muscle damage is less in children than adults.

The magnitude of the protective effect conferred by EC1 against EC2 was similar amongst groups for all dependent variables except for plasma CK activity and myoglobin concentration (Figure 5). The smaller repeated bout effect for plasma CK activity and myoglobin concentration was most likely due to the lack of or small increases in these parameters after EC1 for the pre-pubescent and pubescent groups, and the magnitude of the differences in the repeated bout effect was not necessary very large either (Figure 5). Furthermore, our previous study (Chen et al., 2014) also found that the magnitude of the repeated bout effect was similar among pre-pubescent, pubescent and post-pubescent men. It seems likely that children have similar adaptability to eccentric exercise to adults. In the present study, the same eccentric exercise was repeated 2 weeks later, and the first exercise bout induced some muscle damage even for the pre-pubescent group. However, it has been reported that the magnitude of muscle damage after maximal eccentric exercise is reduced by “non-damaging” low-intensity eccentric contractions (Chen et al., 2012) or maximal isometric contractions at a long muscle length (Chen et al., 2013) performed some days earlier in adults. If this is also case for children (which is most likely but needed to be confirmed), this should be applied for children, even though they are less susceptible to eccentric exercise-induced muscle damage. The mechanisms underpinning the repeated bout effect or the protective effect have not been fully elucidated, although it has been stated that a combination of neural adaptations, muscle-tendon complex behavior changes, extracellular matrix (ECM) structural remodeling, and modified inflammatory responses is involved in the mechanisms (Hyldahl et al., 2017). It is assumed that the mechanisms of the repeated bout effect are similar between adults and children, since the magnitude of the effect was similar between groups (Figure 5). However, it is interesting to investigate whether this is correct.

Some studies reported that the magnitude of muscle damage was less in women than men (Dannecker et al., 2003; Sewright et al., 2008). Since the exercise protocol was not the same between the present and previous studies (Chen et al., 2014), the comparison requires some caution. The magnitude of decrease in maximal concentric strength of the elbow flexors of pre-pubescent, pubescent and post-pubescent groups at 1 day after EC1 was 10, 14, and 25%, respectively in the present study, which was smaller than that shown in the previous study (17, 23, and 32% for pre-pubescent, pubescent and post-pubescent males, respectively; Chen et al., 2014). This may be due to the difference in the exercise protocol (i.e., submaximal vs. maximal), but it is also possible that sex difference in muscle damage already exists during the pre-pubertal period. Previous studies (Tiidus, 2003; Sipaviciene et al., 2013) reported that the sex difference in muscle damage was due to estrogen concentration that could protect plasma membrane damage. However, it does not appear that high estrogen concentration protects muscle damage, since muscle damage was greater for post-pubescent and pubescent groups than pre-pubescent group (Figures 1, 2).

Another factor that may account for the sex-related differences in muscle damage is the force output when normalized to segmental muscle mass. In fact, Blimkie and Sale (1998) showed that specific force was systematically lower in girls than boys during growth, because girls had less capacity to fully activate motor units (Streckis et al., 2007; O'Brien et al., 2010b). O'Brien et al. (2010b) reported that the increases in force and associated muscle-tendon stiffness during growth were smaller in females than males. It seems possible that gender difference in muscle damage already exists already in children. It is also possible that the higher force output in boys results in a higher strain per muscle fiber, which could induce greater muscle damage and lead to more severe symptoms in boys than girls. Previous studies have shown (Falk et al., 2009; Dotan et al., 2012; Ratel and Martin, 2015) that children are less able to recruit or fully utilize their higher-threshold motor units during maximal voluntary isometric contractions of the elbow flexors and extensors when compared with adults. However, further studies are required to compare pre-pubescent, pubescent and post-pubescent males and females for the magnitude of muscle damage by using the same eccentric exercise protocol to examine when sex difference in muscle damage starts to become evident, if sex difference exists.

### Proprioception

Decreases in PS and JRA following EC1 showed that proprioception was negatively affected after unaccustomed eccentric exercise for all age groups; however similar to muscle damage markers discussed above, the decreases were smaller for children than adults, and pre-pubescent than pubescent group (Figure 3A). Paschalis et al. (2010) reported that when muscle damage was induced, proprioception was impaired in young women, speculating that the decreased proprioception following eccentric exercise was related to impaired muscle spindle function due to muscle damage. After EC2, the decreases in proprioception were smaller that those after EC1 for all groups (Figure 3). Thus, it seems likely that the smaller decreases in PS and JRA in children than adults, and pre-pubescent than pubescent group were due to less muscle damage in children than adults, and pre-pubescent than pubescent girls. It has been reported that muscle fascicle length changes are smaller during the second than the first eccentric exercise bout (Lau et al., 2015; Peñailillo et al., 2015). Thus, mechanical strain to muscle spindles could be lower during the second eccentric exercise bout than the first bout. The same principle could be applied to explain why changes in PS and JRA were smaller for children than adults. However, further studies are necessary to investigate how exactly muscle proprioceptors such as muscle spindles and Golgi tendon organs are affected by eccentric contractions during maturation.

### Arterial stiffness

The baseline cfPWV among the three age groups were comparable to those in a previous study (Niboshi et al., 2006). As shown in Figure 4, cfPWV increased after EC1 for all three age groups, but the magnitude of the increase was smaller for children than adults, and pre-pubescent than pubescent girls (Figure 4). Barnes et al. (2010) showed that eccentric contractions of the unilateral elbow flexors increased cfPWV by 5% at 2 days post-exercise for young men. Lin et al. (2017) reported that eccentric exercise of the bilateral knee extensors increased cfPWV by 13% at 2 days post-exercise performed by young healthy men. Barnes et al. (2010) and Lin et al. (2017) speculated that high-tension (mechanical stress) produced during eccentric contractions caused focal disruption of the sarcomere, microvasculature, and/or excitation-contraction coupling apparatus in the skeletal muscle, inducing a subsequent inflammatory response that could affect local and systemic vascular function either directly or indirectly. Choi et al. (2016) explained that significant increases in central arterial stiffness following maximal eccentric exercise was due to a decrease in endothelial function caused by the decreases in endothelium-dependent vasodilation.

The new findings of the present study were that some increases in arterial stiffness were induced after eccentric exercise of the elbow flexors for children, although the magnitude of the increase was smaller than adults, and the second bout of the eccentric exercise resulted in less increases in cfPWV than the first bout for all age groups. Sawabe et al. (2005) documented that the increase in PWV with aging was due to decrease in the elastic properties caused by the degeneration of the arterial wall (decrease in elastin and increase in collagen). Therefore, it seems likely that the smaller increases in cfPWV following eccentric exercise for children than adults were due to less muscle damage for children than adults.

## Conclusions

In summary, the results of the current study showed that the extent of muscle damage, and its effects on proprioception and arterial stiffness changes after eccentric exercise of the elbow flexors were significantly smaller for pre-pubescent girls, followed by pubescent girls than women, but the magnitude of the repeated bout effect was similar among the groups. Together with the previous study (Chen et al., 2014), it is concluded that children are less susceptible to eccentric exercise-induced muscle damage than adults, and the extent of muscle damage increases with maturation status from 9–10 y to 20–25 y. This is probably due to changes in muscles such as muscle fiber composition, muscle and tendon stiffness, muscle spindle and vascular function with growth, but further studies are required to investigate what changes with growth make muscles more susceptible to eccentric exercise-induced muscle damage. These results indicate that children can adapt to eccentric exercise in a similar way to adults. Thus, it is possible to apply the same strategy to introduce eccentric exercise to children such that starting from low-intensity, gradually increasing the intensity and volume based on the progressive overload principal. Further studies are required to investigate the effects of eccentric exercise training on children.

## Author contributions

All authors contributed to the data analysis and interpretation of the data, drafting, and revising the manuscript, and approved the final version of the manuscript. The original study design was made by TC-CC and KN, and discussed with the other authors. M-JL, TC-CC, C-CH, and H-LC performed the data collection.

### Conflict of interest statement

The authors declare that the research was conducted in the absence of any commercial or financial relationships that could be construed as a potential conflict of interest.
